# Assessment of Antioxidants in Selected Plant Rootstocks

**DOI:** 10.3390/antiox9030209

**Published:** 2020-03-03

**Authors:** Samuel Magnus, Filip Gazdik, Naser A. Anjum, Eliska Kadlecova, Zuzana Lackova, Natalia Cernei, Martin Brtnicky, Jindrich Kynicky, Borivoj Klejdus, Tomas Necas, Ondrej Zitka

**Affiliations:** 1 Department of Fruit Science, Faculty of Horticulture, Mendel University in Brno, Valticka 337, 691 44 Lednice, Czech Republic; xmagnus@node.mendelu.cz (S.M.); tomas.necas@mendelu.cz (T.N.); 2 Mendeleum—Institute of Genetics, Mendel University in Brno, Valticka 334, 691 44 Lednice, Czech Republic; filip.gazdik@mendelu.cz (F.G.); xkadleco@node.mendelu.cz (E.K.); 3 Department of Botany, Aligarh Muslim University, Aligarh 202 002, U.P., India; 4 Department of Chemistry and Biochemistry, Mendel University in Brno, Zemedelska 1, CZ-613 00 Brno, Czech Republic; zuzana.lackova@mendelu.cz (Z.L.); cernei.natalia3@gmail.com (N.C.); klejdusb@seznam.cz (B.K.); 5 Central European Institute of Technology, Mendel University in Brno, Zemedelska 1, CZ-613 00 Brno, Czech Republic; 6 Department of Agrochemistry, Soil Science, Microbiology and Plant Nutrition, Mendel University, 613 00 Brno, Czech Republic; Martin.Brtnicky@seznam.cz; 7 Institute of Chemistry and Technology of Environmental Protection, Brno University of Technology, Faculty of Chemistry, Purkynova 118, 621 00 Brno, Czech Republic; 8 BIC Brno, Technology Innovation Transfer Chamber, 612 00 Brno, Czech Republic; jindrak@email.cz

**Keywords:** phenolic compounds, flavonoid compounds, procyanidin compounds, catechin compounds, LC/MS, *Sorbus domestica*, rootstocks of plants

## Abstract

The service tree (*Sorbus domestica*) is a wild fruit tree with immense medicinal and industrial value. This study aimed at determining the four major groups of antioxidants (flavonoids, phenolic acids and aldehydes, catechin and procyanidin) in rootstocks of *Crataegus laevigata* (genotypes O-LE-14 and O-LE-21), *Aronia melanocarpa* (genotypes O-LE-14 and O-LE-21), *Chaenomeles japonica* (genotype O-LE-9) and *Cydonia oblonga* (BA 29) (genotypes O-LE-14 and O-LE-21). Hyperoside (Quercetin 3-D-galactoside) was the most abundant flavonoid compound, since its average content in the rootstocks of *Crataegus laevigata* (O-LE-21) was 180.68 ± 0.04 μg·g^−1^. Dihydrokaempherol was the least frequently found flavonoid compound, with an average concentration of 0.43 ± 0.01 μg·g^−1^ in all the rootstocks of plants considered in this study. Among the phenolic compounds, the most represented one was protocatechuic acid, with 955.92 ± 10.25 μg·g^−1^ in the rootstocks of *Aronia melanocarpa* (O-LE-14). On the other hand, the least represented *p*-Coumaric acid exhibited the average concentration of 0.34 ± 0.01 μg·g^−1^ in the plant rootstocks. Epicatechin was the most abundant catechin compound, with a content of 3196.37 ± 50.10 μg·g^−1^ in the rootstocks of *Aronia melanocarpa* (O-LE-14). The lowest represented catechin compound was epigallocatechin, with the average concentration of 0.95 ± 0.08 μg·g^−1^ in the screened plant rootstocks. From the procyanidin compounds, the most abundant one was procyanidin b2 in the rootstocks of *Crataegus laevigata* (O-LE-14), with a concentration of 5550.40 ± 99.56 μg·g^−1^. On the contrary, procyanidin a2, with an average concentration of 40.35 ± 1.61 μg·g^−1^, represented the least frequent procyanidin compound in all the plant rootstocks screened herein.

## 1. Introduction

Plants are known to contain flavonoids, phenolic acids and aldehydes, along with catechin and procyanidin derivatives. These compounds play a significant role in the growth, metabolism and stress tolerance of plants [1] and are known to contribute equally to human health [2,3,4,5,6,7]. In humans, phenolic derivatives display well established antioxidant activities and contribute to various cellular functions [3,4,8,9,10,11,12,13,14]. Due to their low bioavailability, humans can obtain these compounds directly through the consumption of plants, where various phenolic compounds participate in the process of cell division, development and differentiation [15]. These are important in terms of protection against herbivores, owing to their bitter taste and toxic potential. The concentration of particular phenolic compounds and their subsequent toxicity is highly variable, and depend on the particular plant part, its growth stage and the season of the year [16].

Phenolic acids can be divided into two groups based on their solubility in water: soluble and non-soluble. The water soluble group of phenolic acids are present in vacuoles, whereas the non-soluble ones occur in cell walls [17]. They protect plants against herbivores and pathogens, and are also involved in pollination, pigmentation, growth and development [18]. High amounts of these simple phenols can be found in fruits and vegetables [19]. In addition to protecting DNA against oxidative stress, flavonoids inactivate enzymes responsible for carcinogenic activity by acting as anticarcinogens [20,21,22]. Additionally, flavonoids can also possess antivirotic, antibacterial and antimycotic activity [5,6]. These substances also have the ability to damage pathogen spores [7]. In plants, flavonoid compounds are involved in crucial biochemical and physiological processes, and are also significant for numerous cultural species and rootstocks [23,24,25,26]. Catechins are colorless, odorless, soluble substances with low molecular mass. They are among the main substances causing the incompatibility with the rootstocks [27]. Catechins are also responsible for enzymatic browning [28]. Moreover, catechins lower the oxidation of linoleic acid, which is known for protecting the lipidic membrane and exhibiting its anti-oxidative effect [29,30]. On the other hand, procyanidins can be found in fruits, vegetables and other foods [31]. Thus, all the above-mentioned compoundsare beneficial to humans, andalso are important in horticultural practice, based on their biochemistry.

Grafting is a method of deliberating vegetative crop reproduction that is commonly used in horticultural practice to maintain and reproduce cultivars of plants that have beneficial properties for growers and for genetic engineering. This method is commonly used in agriculture to increase the yields, modify the industrial production and also in improving stress tolerance. However, the underlying mechanisms fostering the productivity of certain specific combinations of grafts remain largely unknown [32]. When a tree becomes grafted, the healing of the wound starts, which is a complex biochemical process involving an immediate reaction of the wound, callus formation, the creation of new tissue and the formation of a vascular system between the rootstock and the variety [33,34]. The tissue, organ and whole plant level of flavonoid, phenolic acids and aldehydes, catechin and procyanidin compounds can be modulated through several approaches, including grafting [35,36,37,38].

The detection of phenolic compounds at the initial growth stages following grafting is of particular interest, as these may block the vascular connection between the vascular cambium tissues of the rootstock and scion species [39]. Additionally, the accumulation of phenols (anthocyanins, flavanones, p-coumaric acid and hydroxybenzoic acid) has been associated with reduced graft compatibility at both early and late stages in apricot [39] and peach grafted plants [40]. Musacchi et al. showed that the compounds that most closely respond to these requisites are the flavanol monomers epicatechin and catechin and the dimer procyanidin B_2_ with epicatechin, evincing the highest interface concentration increase in quince-in-compatible unions [28]. These data confirm the findings reported for apricot by Errea et al. [41], who observed an increase of catechins and proanthocyanidins under stress due to graft incompatibility. However, literature is lacking on the studies unveiling the major insights into the composition of flavonoid, phenolic acids and aldehydes, catechin and procyanidin compounds, including the combination of various rootstocks and genotypes for *S. domestica*.

Accordingly, this study aimed at determining the selected flavonoid, phenolic acids and aldehydes, catechin and procyanidin compounds in seven grafting-modified kinds of rootstocks of *Sorbus domestica, Crataegus laevigata* (genotypes O-LE-14 and O-LE-21), *Aronia melanocarpa* (genotypes O-LE-14 and O-LE-21), *Chaenomeles japonica* (genotypes O-LE-9) and *Cydonia oblonga* (BA 29) (genotypes O-LE-14 and O-LE-21), with one control (*S. domestica*).

## 2. Materials and Methods

### 2.1. Chemicals

The chemicals used in different procedures during the present study were purchased from Sigma-Aldrich (St. Louis, MO, USA) in ACS purity, unless noted otherwise. 

### 2.2. Plants

In this study, seven varieties of rootstocks potentially suitable for *S. domestica* (as *Crataegus laevigata* (genotypes O-LE-14 and O-LE-21), *Aronia melanocarpa* (genotypes O-LE-14 and O-LE-21), *Chaenomeles japonica* (genotypes O-LE-9) and *Cydonia oblonga* (BA 29) (genotypes O-LE-14 and O-LE-21)) with the control (seedlings of *Sorbus domestica* L.) were used. These rootstocks were planted in October 2017 (in Lednice, a village in South Moravia in the Czech Republic), grafted in February/March 2018 and harvested in July 2018. After the samples had been taken out of the soil, the bark was manually removed from the graft union part and the phloem was instantly deep-frozen in a friction bowl with temperature −80 °C. Subsequently, the samples were crushed by friction in the presence of liquid nitrogen (−196°C).

### 2.3. Preparation of the Plant Samples for Analyzing Flavonoid, Phenolic Acids and Aldehydes, Catechin and Procyanidin Compounds

In this experiment, seven kinds of rootstocks of *Sorbus domestica* with the control (*S. domestica*) were used. Methanol (80%, *v*/*v*) was used in the extraction of the flavonoid, the phenolic acids and aldehydes and the catechin and procyanidin compounds from the rootstocks of *S. domestica*. The samples were subjected to lyophilization for 24 h, 0.014 mBar vacuum and −55 °C (Lyophilizer, Labconco, Kansas City, Missouri, USA). An equal weight (20 mg) of samples was taken from each of the seven kinds of rootstocks of *S. domestica* with the control (Analytical Balance, EP 240A, Precisa, Vienna, Austria). The samples were homogenized in a friction bowl with 1.0 mL of 80% methanol, and 0.05 to 0.1 g of sea sand, until evaporation. The homogenization was repeated twice. Thereafter, the samples were vortexed (Vortex Mixers, VELP Scientifica, Usmate Velate MB, Italy) for 1–2 min and were subsequently centrifuged at 25,000 rpm and 16 °C for 15 min (Centrifuge Z326K, Hermle, Gosheim, Germany). Later, each sample was filtered through a filter (LUT Syringe Filters Nylon, LABICOM s.r.o., Olomouc, Czech Republic). Finally, the samples were pipetted out (400 µL) and analyzed using LC/MS. The results have been recalculated per 1.0 g of plant tissue.

### 2.4. Analysis of the Plant Sample-Extracts Using LC/MS

To determine the selected flavonoid, phenolic acids and aldehydes, and catechin and procyanidin compounds, a high-performance liquid chromatograph (HPLC Agilent 1200 Series) with a triple quadrupole and the mass detector (6460 Triple Quad) LC/MS equipped with ESI ionization were used. For the separation of the flavonoid, the phenolic acids and aldehydes and the catechin and procyanidin compounds, a Zorbax EC 18 column of 50 × 3.0 mm and a particle size of 2.7 μm was used prior to analyzing the compounds of interest. The measured concentration was the average of three measurements (injections) for each sample of triplicate. The acquired data between triplicates varied within RSD 5%.

#### 2.4.1. Separation of Flavonoid Compounds

The column was held at 60 °C. The mobile A phase consisted of 100% methanol, whereas the mobile B phase was 0.2% acetic acid. The flow rate of the mobile phase was kept at 0.7 mL·min^−1^. The compounds were eluted with a linear upward gradient: 0 min (90% B), 2 min (40% B), 4 min (0% B) and 6 min (90% B). The triple quadrupole mass detector was operated in the negative mode. Gas (nitrogen) temperature was kept at 350 °C, the gas flow rate was set to 13 L·min^−1^, the pressure nebulizer had a value of 50 psi, the temperature of the focusing gas was 400 °C, the flow rate of the focusing gas was set at 12 L·min^−1^ and the voltage on the capillary tube amounted to 4000 V (Table 1)

#### 2.4.2. Separation of Phenolic Acids and Aldehydes 

The column was held at 45 °C. The mobile A phase consisted of 100% methanol, and the mobile B phase was 0.2% acetic acid. The flow rate of the mobile phase was kept at 0.6 mL·min^−1^. The compounds were eluted with a linear upward gradient: 0.00 min (82% B), 0.17 min (82% B), 0.51 min (70% B), 1.70 min (45% B), 4.00 min (45% B) and 6.00 min (82% B). The triple quadrupole mass detector was operated in negative mode. The gas (nitrogen) temperature was kept at 300 °C, the gas flow rate was set to 12 L·min^−1^, the pressure nebulizer had a value of 60 psi, the temperature of the focusing gas was 300 °C, the flow rate of the focusing gas was set at 11 L·min^−1^ and the voltage on the capillary tube amounted to 3500 V (Table 2).

#### 2.4.3. Separation of Catechin and Procyanidin Compounds

The column was held at 45 °C. The mobile A phase consisted of 100% methanol, and the mobile B phase was 0.2% acetic acid. The flow rate of the mobile phase was kept at 0.6-0.7 mL·min^−1^. The compounds were eluted with a linear upward gradient: 0.00 min (85% B), 0.17 min (85% B), 0.51 min (75% B), 1.70 min (70% B), 4.00 min (70% B) and 6.00 min (85% B). The triple quadrupole mass detector was operated in the negative mode. The gas (nitrogen) temperature was 300 °C, the gas flow rate was set to 12 L·min^−1^, the pressure nebulizer had a value of 45 psi, the temperature of the focusing gas was 250 °C, the flow rate of the focusing gas was set at 11 L·min^−1^ and the voltage on the capillary tube amounted to 3500 V (Table 3).

### 2.5. Statistics Methodology

The data were processed using MICROSOFT EXCEL^®^ (Microsoft, Redmond, WA, USA) and STATISTICA CZ Version 12.0 (StatSoft CR s.r.o., Prague, Czech Republic). The data are expressed as mean ± standard deviation (S.D.), unless otherwise noted (EXCEL). The statistical significance of the measured data was determined using STATISTICA CZ. The Anderson–Darling test was used to test the normality of the data. Differences with *p* < 0.05 were considered significant and were determined by using a one-way ANOVA test and a post-hoc Dunnett’s test, which was applied as a means of comparison to the control group. Moreover, for the exploratory data analysis (EDA) cluster analysis, the principle component analysis (PCA) and the correlation were done.

## 3. Results

### 3.1. LC/MS-Based Profile of the Test Plant Extracts

A LC/MS analysis was performed to determine different flavonoid, phenolic, catechin and procyanidin compounds in selected rootstocks of different plants. The determination of the occurrence and content of flavonoid, phenolic acids and aldehydes, catechin, and procyanidin compounds was done using high-performance liquid chromatography with mass detection. 

#### 3.1.1. Occurrence and Contents of Selected Flavonoid Compounds

**Figure 1:**
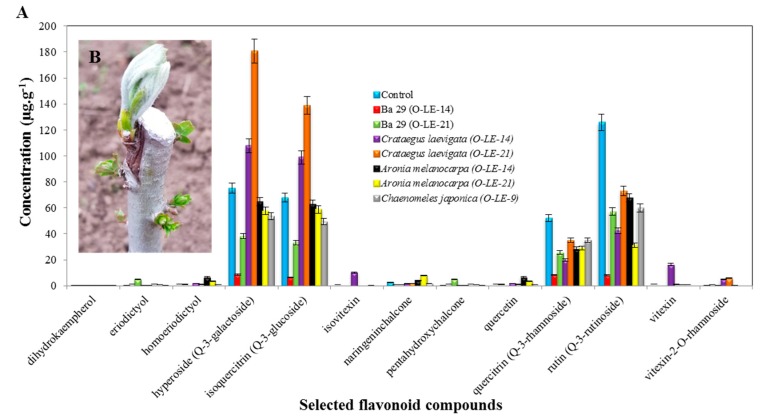
(**A**) Determination of the concentration of selected flavonoid compounds in the extract from the rootstocks of plants; (**B**) demonstration of the cleavage site.

Hyperoside (Q-3-galactoside) was the most abundant flavonoid compound, followed by isoquercitrin (Q-3-glucoside) and rutin (Q-3-rutinoside) in selected rootstocks of plants studied herein (Figure 1).

##### (A) Hyperoside (Q-3-galactoside)

The most represented flavonoid compound was hyperoside (Q-3-galactoside). *Crataegus laevigata* (O-LE-21), *Crataegus laevigata* (O-LE-14), the control, *Aronia melanocarpa* (O-LE-14), *Aronia melanocarpa* (O-LE-21), *Chaenomeles japonica* (O-LE-9), BA 29 (O-LE-21) and BA 29 (O-LE-14) exhibited 180.68 ± 0.04 μg·g^−1^, 107.72 ± 0.08 μg·g^−1^, 75.30 ± 0.04 μg·g^−1^, 64.78 ± 0.12 μg·g^−1^, 57.62 ± 0.03 μg·g^−1^, 53.75 ± 0.03 μg·g^−1^, 38.20 ± 0.07 μg·g^−1^ and 8.60 ± 0.05 μg·g^−1^ of hyperoside (Q-3-galactoside) respectively. A higher concentration of hyperoside compared to the control was recorded with *Crataegus laevigata* (O-LE-14) and *Crataegus laevigata* (O-LE-21). Lower concentrations of hyperoside compared to the control were observed in other rootstocks of plants.

##### (B) Isoquercitrin (Q-3-glucoside)

The second most represented flavonoid compound was isoquercitrin (Q-3-glucoside), with a concentration of 138.87 ± 0.05 μg·g^−1^ in *Crataegus laevigata* (O-LE-21), 98.84 ± 0.04 μg·g^−1^ in *Crataegus laevigata* (O-LE-14), 67.97 ± 0.03 μg·g^−1^ in the control, 62.90 ± 0.04 μg·g^−1^ in *Aronia melanocarpa* (O-LE-14), 58.87 ± 0.03 μg·g^−1^ in *Aronia melanocarpa* (O-LE-21), 49.46 ± 0.03 μg·g^−1^ in *Chaenomeles japonica* (O-LE-9), 33.09 ± 0.01 μg·g^−1^ in BA 29 (O-LE-21) and 6.58 ± 0.03 μg·g^−1^ in BA 29 (O-LE-14). A higher concentration of isoquercitrin compared to the control was recorded with *Crataegus laevigata* (O-LE-14) and *Crataegus laevigata* (O-LE-21). Lower concentrations of isoquercitrin compared to the control were observed in other rootstocks of plants.

##### (C) Rutin (Q-3-rutinoside)

The third most represented flavonoid compound was rutin (Q-3-rutinoside), with a concentration of 125.69 ± 0.03 μg·g^−1^ in the control, 73.19 ± 0.05 μg·g^−1^ in *Crataegus laevigata* (O-LE-21), 67.69 ± 0.04 μg·g^−1^ in *Aronia melanocarpa* (O-LE-14), 59.87 ± 0.04 μg·g^−1^ in *Chaenomeles japonica* (O-LE-9), 57.18 ± 0.03 μg·g^−1^ in BA 29 (O-LE-21), 42.39 ± 0.04 μg·g^−1^ in *Crataegus laevigata* (O-LE-14), 31.21 ± 0.04 μg·g^−1^ in *Aronia melanocarpa* (O-LE-21) and 8.10 ± 0.10 μg·g^−1^ in BA 29 (O-LE-14). Compared to the control, lower contents of rutin were observed in all the rootstocks of plants. The least most represented flavonoid compound was quercitrin (Q-3-rhamnoside). The control, *Chaenomeles japonica* (O-LE-9), *Crataegus laevigata* (O-LE-21), *Aronia melanocarpa* (O-LE-21), *Aronia melanocarpa* (O-LE-14), BA 29 (O-LE-21), *Crataegus laevigata* (O-LE-14) and BA 29 (O-LE-14) exhibited, respectively, 52.33 ± 0.04 μg·g^−1^, 35.22 ± 0.07 μg·g^−1^, 35.06 ± 0.05 μg·g^−1^, 29.29 ± 0.03 μg·g^−1^, 28.59 ± 0.03 μg·g^−1^, 25.66 ± 0.02 μg·g^−1^, 19.91 ± 0.04 μg·g^−1^ and 8.41 ± 0.10 μg·g^−1^. Lower concentrations of quercitrin compared to the control were observed in all the rootstocks of plants. 

#### 3.1.2. Contents of Phenolic Acids and Aldehydes

**Figure 2:**
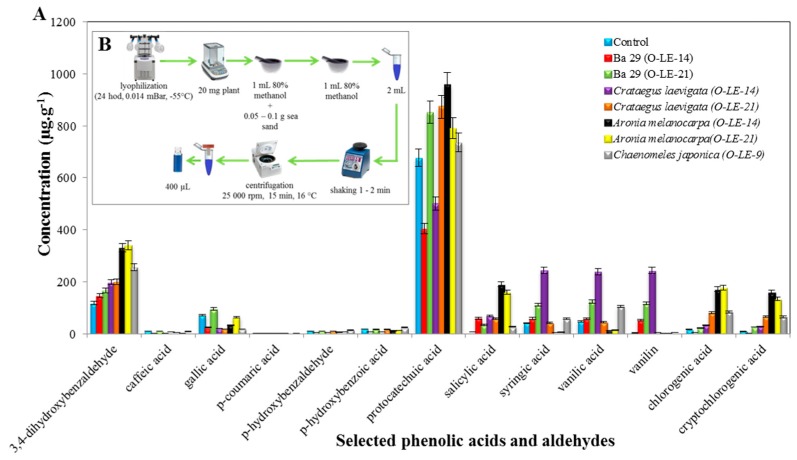
(**A**) Determination of the concentration of selected phenolic acids and aldehydes in the extract from the rootstocks of plants; (**B**) scheme of preparation of the sample for the LC/MS analysis.

Protocatechuic acid was the most frequent phenolic compound, followed by 3,4-Dihydroxybenzaldehyde, syringic acid, vanilic acid and vanillin in selected rootstocks of plants (Figure 2A). 

##### (A) Protocatechuic acid

The predominant phenolic compound was protocatechuic acid, occurring with a concentration of 955.92 ± 10.25 μg·g^−1^ in *Aronia melanocarpa* (O-LE-14), 872.77 ± 9.88 μg·g^−1^ in *Crataegus laevigata* (O-LE-21), 851.34 ± 11.25 μg·g^−1^ in BA 29 (O-LE-21), 790.29 ± 10.00 μg·g^−1^ in *Aronia melanocarpa* (O-LE-21), 735.19 ± 9.99 μg·g^−1^ in *Chaenomeles japonica* (O-LE-9), 676.79 ± 8.95 μg·g^−1^ in the control, 500.65 ± 12.51 μg·g^−1^ in *Crataegus laevigata* (O-LE-14) and 403.66 ± 9.85 μg·g^−1^ in BA 29 (O-LE-14). Compared to the control, a higher concentration of protocatechuic acid was recorded in *Aronia melanocarpa* (O-LE-14), *Crataegus laevigata* (O-LE-21), BA 29 (O-LE-21), *Aronia melanocarpa* (O-LE-21) and *Chaenomeles japonica* (O-LE-9). A lower concentration of protocatechuic acid occurred in other plant rootstocks when compared to the control.

##### (B) 3,4-Dihydroxybenzaldehyde

The second most represented phenolic compound was 3,4-Dihydroxybenzaldehyde, exhibiting a concentration of 339.96 ± 8.12 μg·g^−1^ in *Aronia melanocarpa* (O-LE-21), 330.14 ± 10.01 μg·g^−1^ in *Aronia melanocarpa* (O-LE-14), 255.26 ± 12.11 μg·g^−1^ in *Chaenomeles japonica* (O-LE-9), 199.20 ± 8.55 μg·g^−1^ in *Crataegus laevigata* (O-LE-21), 197.32 ± 8.62 μg·g^−1^ in *Crataegus laevigata* (O-LE-14), 166.28 ± 7.99 μg·g^−1^ in BA 29 (O-LE-21), 146,16 ± 8.23 μg·g^−1^ in BA 29 (O-LE-14) and 117.58 ± 6.88 μg·g^−1^ in the control. Notably, compared to the control, lower concentrations of 3,4-Dihydroxybenzaldehyde were observed in all the rootstocks of plants in the present study.

##### (C) Syringic acid, vanilic acid and vanilin

Interestingly, syringic acid, vanilic acid and vanilin occurred in higher concentrations in the extract of *Crataegus laevigata* (O-LE-14) rootstocks. In the case of syringic acid, the control exhibited 40.65 ± 1.21 μg·g^−1^), whereas 243.66 ± 7.93 μg·g^−1^ of syringic acid occurred in *Crataegus laevigata* (O-LE-14) rootstocks. The concentration of vanilic acid was very similar to that of syringic acid (238.90 ± 9.68 μg·g^−1^), and the control showed 47.94 ± 3.41 μg·g^−1^ of syringic acid. However, the concentration of vanilin, when compared to the control, was surprising. In the extract of rootstocks of *Crataegus laevigata* (O-LE-14), 242.78 ± 13.21 μg·g^−1^ of vanillin was observed, whereas its concentration in the control was only 2.73 ± 0.01 μg·g^−1^. The other flavonoid compounds revealed concentrations not exceeding 189 μg·g^−1^. Figure 2B shows the major steps followed during the preparation of samples for the LC/MS analysis. The experimental details can be found in Section 2.

#### 3.1.3. Contents of Selected Catechin and Procyanidin Compounds

**Figure 3:**
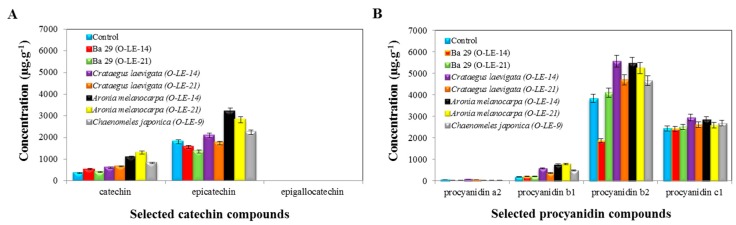
(**A**) Determination of the concentration of selected catechin compounds in the extract from the rootstocks of plants; (**B**) Determination of the concentration of selected procyanidin compounds in the extract from the rootstocks of plants.

Catechin and procyanidin compounds were also determined in selected rootstocks of plants. Catechin, epicatechin and epigallocatechin were selected among the catechins for analysis (Figure 3A).

##### (A) Catechin Compounds

a.1. Epicatechin

The most often-represented catechin compound in the chosen rootstocks of plants was epicatechin. Epicatechin exhibited concentrations of 3196.37 ± 50.10 μg·g^−1^, 2806.78 ± 45.12 μg·g^−1^, 2243.11 ± 60.21 μg·g^−1^, 2085.88 ± 49.62 μg·g^−1^, 1800.49 ± 56.88 μg·g^−1^, 1741.14 ± 66.55 μg·g^−1^, 1562.60 ± 63.23 μg·g^−1^ and 1353.07 ± 49.99 μg·g^−1^ in *Aronia melanocarpa* (O-LE-14), *Aronia melanocarpa* (O-LE-21), *Chaenomeles japonica* (O-LE-9), *Crataegus laevigata* (O-LE-14), the control, *Crataegus laevigata* (O-LE-21), BA 29 (O-LE-14) and BA 29 (O-LE-21), respectively. A higher concentration of epicatechin compared to the control was recorded with *Aronia melanocarpa* (O-LE-14), *Aronia melanocarpa* (O-LE-21), *Chaenomeles japonica* (O-LE-9) and *Crataegus laevigata* (O-LE-14). On the other hand, compared to the control, lower concentrations of epicatechin were observed in other rootstocks of plants.

a.2. Catechin

Catechin was another highly represented compound. The lowest concentration of catechin was measured in the control (361.62 ± 11.65 μg·g^−1^). However, 1302.59 ± 48.12 μg·g^−1^, 1093.79 ± 60.89 μg·g^−1^ and 821.44 ± 33.62 μg·g^−1^ concentrations of catechin were found in *Aronia melanocarpa* (O-LE-21), *Aronia melanocarpa* (O-LE-14) and *Chaenomeles japonica* (O-LE-9), respectively. For the other rootstocks of plants, the values were almost equal to the average 552.59 ± 35.22 μg·g^−1^. Catechin is considered to be the main cause of delayed lack of affinity.

a.3. Epigallocatechin

Very low concentrations of epigallocatechin were measured in all the rootstocks of plants tested herein. The control exhibited a concentration value of 0.84 ± 0.01 μg·g^−1^. Compared to the control, the highest concentration of epigallocatechin was detected in *Aronia melanocarpa* (O-LE-21) (1.23 ± 0.03 μg·g^−1^) and *Crataegus laevigata* (O-LE-14) (1.23 ± 0.04 μg·g^−1^), whereas BA 29 (O-LE-21), compared to the control, exhibited the lowest concentration (0.48 ± 0.01 μg·g^−1^). The other rootstocks of plants exhibited concentrations lower than 1.00 ± 0.01 μg·g^−1^.

##### (B) Procyanidin Compounds

b.1. Procyanidin b2

Among the procyanidin compounds, procyanidin b2 was the most represented, followed by procyanidin c1 and procyanidin a2 (Figure 3B). The highest concentration of this compound was detected in *Crataegus laevigata* (O-LE-14) (5550.40 ± 99.56 μg·g^−1^), followed by 5452.11 ± 99.10 μg·g^−1^ of procyanidin b2 in *Aronia melanocarpa* (O-LE-14), 5238.47 ± 89.92 μg·g^−1^ in *Aronia melanocarpa* (O-LE-21), 4690.26 ± 87.55 μg·g^−1^ in *Crataegus laevigata* (O-LE-21), 4656.36 ± 88.21 μg·g^−1^ in *Chaenomeles japonica* (O-LE-9), 4093.37± 89.99 μg·g^−1^ in BA 29 (O-LE-21), 3822.64 ± 76.88 μg·g^−1^ in the control and 1852.16 ± 65.43 μg·g^−1^ of procyanidin b2 in BA 29 (O-LE-14). All the rootstocks of plants used herein had procyanidin b2 concentrations exceeding that of the control, except for the rootstocks of BA 29 (O-LE-14).

b.2. Procyanidin c1

The second most represented compound from the procyanidin group was procyanidin c1. However, there were only statistically insignificant differences between the observed concentrations. The average procyanidin c1 concentration was 2 619.68 ± 101.92 μg·g^−1^. The concentration of procyanidin c1 has not yet been described in the grafting spot. As for procyanidin b1, the concentration was higher in all the tested rootstocks of plants compared to the control (181.69 ± 21.10 μg·g^−1^). Procyanidin b1 concentrations of 779.73 ± 29.92 μg·g^−1^, 740.95 ± 39.10 μg·g^−1^, 566.55± 37.55 μg·g^−1^, 475.10 ± 10.21 μg·g^−1^, 364.93 ± 11.55 μg·g^−1^, 200.76 ± 9.56 μg·g^−1^ and 196.42 ± 8.99 μg·g^−1^ were observed in *Aronia melanocarpa* (O-LE-21), *Aronia melanocarpa* (O-LE-14), *Crataegus laevigata* (O-LE-14), *Chaenomeles japonica* (O-LE-9), *Crataegus laevigata* (O-LE-21), BA 29 (O-LE-14) and BA 29 (O-LE-21), respectively.

b.3. Procyanidin a2

Compared to the control, the lowest concentration measured in the procyanidin group of compounds was that of procyanidin a2. Compared to the control, its highest concentration was measured in *Crataegus laevigata* (O-LE-14) (68.48 ± 4.25 μg·g^−1^). The concentration of the other rootstocks of plants used was as follows: 56.11 ± 5.88 μg·g^−1^ in *Crataegus laevigata* (O-LE-21), 35.61 ± 1.25 μg·g^−1^ in *Aronia melanocarpa* (O-LE-14), 32.55 ± 1.99 μg·g^−1^ in *Chaenomeles japonica* (O-LE-9), 32.21 ± 1.25 μg·g^−1^ in BA 29 (O-LE-21), 28.5 ± 0.34 μg·g^−1^ in *Aronia melanocarpa* (O-LE-21) and 24.26 ± 1.85 μg·g^−1^ in BA 29 (O-LE-14). When compared to the control, a higher concentration of procyanidin a2 was recorded with *Crataegus laevigata* (O-LE-21) and *Crataegus laevigata* (O-LE-14). Compared to the control, a lower concentration of procyanidin a2 was observed in the other plant rootstocks.

### 3.2. Statistical Analysis

The Anderson–Darling test revealed that all data had a normal distribution. The statistical significance of the differences between the control sample and the other samples, including the majority of anti-oxidative compounds, was tested at *p* < 0.001, employing a one-way ANOVA test and a post-hoc Dunnett’s test (Table S1). In two cases, the significance level was lower (*p* < 0.050), but the control group was still significantly different. Finally, no significant differences were found for quercetin between the control sample and BA 29 (O-LE-14) (*p* < 0.322); the same is true for dihydrokaempferol between the control sample and BA 29 (O-LE-21) (*p* < 1.000), *Crataegus laevigata* (O-LE-14) (*p* < 0.216) and *Aronia melanocarpa* (O-LE-14) (*p* < 0.819).

**Figure 4:**
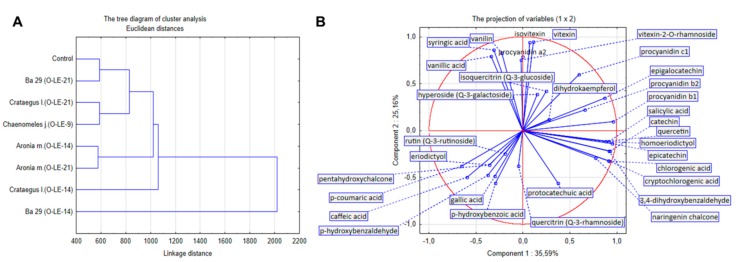
(**A**) The tree diagram of cluster analysis for 8 groups (samples) based on 33 variables (anti-oxidative compounds). Single-linkage clustering with Euclidean distances was used; (**B**) the Principle Component Analysis (PCA) projection of variables (anti-oxidative compounds) into a plane consisted of PCA components 1 and 2. The PCA component 1 represents 35.59% of the total data variability. The component 2 represents 25.16% of the total data variability. The data for statistics are shown in the supplementary material (Tables S1 and S2).

Regarding the samples, the exploratory data analysis (EDA) revealed the most similar sample to the control sample, based on the cluster analysis (Figure 4A), to be BA 29 (O-LE 21). From this point of view, BA 29 (O-LE-14) was the sample that was least similar to the control. On the other hand, the pairs BA 29 (O-LE-21) and BA 29 (O-LE-14), and *Crataegus laevigata* (O-LE-21) and *Crataegus laevigata* (O-LE-14) were not so similar according to the cluster analysis (Figure 4A). Regarding the anti-oxidative compounds, the PCA analysis (Figure 4B) revealed the major four main groups of compounds with good in-group correlations. The closer the vectors of the compounds were in the projection (Figure 4B), the more significant was the correlation between the compounds. The first group consisted of p-coumaric acid, caffeic acid, eriodictyol, pentahydroxychalcone, p-hydroxybenzaldehyde, rutin (Q-3-rutinoside), gallic acid and p-hydroxybenzoic acid. The second group consisted of naringenin chalcone, chlorogenic acid, cryptochlorogenic acid, 3,4-Dihydroxybenzaldehyde, catechin, epicatechin, homoeriodictyol, quercetin and salicylic acid. The third group consisted of vitexin, isovitexin and procyanidin a2. The fourth group consisted of vanilin, syringic acid and vanillic acid. One compound, protocatechuic acid, was found to be distant from the other compounds and groups of compounds, and therefore it did not exhibit a correlation with the other compounds. This was also confirmed in the correlation matrix (Table S2).

## 4. Discussion

Generally, antioxidants have not yet been deeply investigated in connection with the affinity in grafted woods. Hudina et al. reported arbutin as the most abundant phenolic compound in the phloem above and below the graft union, followed by procyanidin B_1_ and chlorogenic acid [27]. Assuncao et al. considered gallic and sinapic acids as the markers of graft/scion compatibility [42]. The authors identified high concentrations of gallic and sinapic acids together with catechin as the cause of decreased affinity. A lower abundance in gallic acid, sinapic acid and catechin in the more compatible combination could be related to a lesser oxidative stress environment of the grafts, consequently promoting a better development of the graft union. Generally, the concentration of flavanols (particularly epicatechin) decreases at the graft interface compared to the surrounding woody tissues. Presumably, the wood has a high concentration of flavanols, which gets diluted as the callus cells develop [43].

The research carried out by Canas et al. [44] on grapevine, among other things, showed that catechin, epicatechin, ferulic acid and caffeic acid seem to have an important involvement in incompatibility, owing to the different content between graft partners, with higher accumulation above the graft union. Other authors highlighted that a quantitative difference in the phenolic compounds produced by heterospecific grafts may result in metabolic dysfunctions between the cells of the scion–rootstock in the graft union [28].

### 4.1. An Occurrence and Contents of Selected Flavonoid Compounds

According to Hudina et al. [27], a higher concentration of hyperoside (Q-3-galactoside), isoquercitin (Q-3-glucoside), quercetine (Q-3-rhamnoside) and rutine (Q-3-rutinoside) in pear rootstock tissues bellow the graft union may indicate incompatibility between the graft and the scion. Although the samples of the current study were taken directly from the graft union, higher concentrations of hyperoside (Q-3-galactoside) and isoquercitine (Q-3-glucoside) were measured at both *C. leavigata* variants (O-LE-14, O-LE-21) when compared to the control (good affinity). In both *C. leavigata* variants, the visible incompatibility was not observed, but future disaffinity cannot be ruled out. On the other hand, in variants with visible disaffinity (*Chaenomeles japonica* (O-LE-9)) and both *Aronia melanocarpa* (O-LE-14 and O-LE-21), the concentrations of these flavonoids did not exceed those of the control. In quercitine (Q-3-rhamnoside) and rutin (Q-3-rutinoside), none of the variants exceeded the concentration of the control, suggesting that they are not signaling an incompatibility between the service tree and the tested rootstocks.

For the rest of the flavonoids, to the best of our knowledge, no information is available in connection with the disaffinity of incompatible scions and rootstocks. However, among these, the concentrations of naringenin chalocone, quercetin and homoeriodictyol, which are widespread in other species [45,46,47], were increased in both variants of *Aronia melanocarpa*, the incompatible rootstocks.

### 4.2. An Occurrence and Contents of Selected Phenolic Acids and Aldehydes

The variants with incompatible rootstocks (*Aronia melanocarpa* (O-LE-14, O-LE-21) and *Chaenomeles japonica* (O-LE-9)) had the highest concentrations of 3,4-Dihydroxybenzaldehyde, salicylic acid, chlorogenic acid and cryptochlorogenic acid compared to the control. High chlorogenic acid concentration was previously reported as the signal of disaffinity in pear trees [27], which is in agreement with our results.

P-coumaric acid was analyzed in rootstock affinity tests in the work of Usenik et al. [48], where high amounts of this acid were accumulated in apricot scions when disaffinity occurred. In the present study, almost no p-coumaric acid was measured in all variants, which in turn suggested that p-coumaric acid does not play a role in the disaffinity of *Sorbus domestica*.

Based on the results, the highest concentrations at all rootstocks were measured for protocatechuic acid, for which higher concentrations than the control were measured in incompatible *Aronia melanocarpa* (O-LE-14, O-LE-21) and *Chaenomeles japonica* (O-LE-9), but also in one out of two compatible variants: BA29 (O-LE-21) and *Crataegus laevigata* (O-LE-21). It seems that protocatechuic acid does not affect or signal the compatibility of the graft and the rootstock. The same could be stated for vanilin, vanilic and syringic acid, where the highest concentrations were observed at compatible rootstocks (Figure 2).

### 4.3. An Occurrence and Contents of Selected Catechin and Procyanidin Compounds

Epicatechin and catechine are well known flavonoids, which increase when disaffinity occurs [27,28,41,45]. Our results are in accordance with this information, as incompatible rootstocks of *Aronia melanocarpa* of both variants and *Chaenomeles japonica* had the highest concentration of these substances. Although higher concentrations of catechine and epicatechine were measured in some of the compatible rootstock variants (Figure 3a) when compared to the control, they were not as high as those of incompatible rootstocks. This effect, together with a higher concentration of epicatechin than catechin in tissues, which was observed in the present study, was described by Musacchi [28]. On the other hand, procyanidin b1 and b2 are potentially involved in a graft incompatibility in pear trees [27]. In our study, the highest concentrations of both procyanidins were measured in incompatible *Aronia melanocarpa* variants, *Chaenomeles japonica* (Figure 3B), which proves their incompatibility. However, high concentrations - higher than those of the control were also measured in compatible *Crataegus laevigata* variants (Figure 3B). Apart from the results of isoquercitine (Q-3-glucoside) and hyperoside (Q-3-galactoside) discussed above, there is a suspicion that *Crateagus leavigata* will show the incompatibility symptoms in next years. Procyanidin a2 and c1 did not show any pattern of signaling incompatibility.

## 5. Conclusions

This study has presented the results of the pilot analysis of the major flavonoids, phenolic acids and aldehydes, catechin and procyanidin compounds in the selected rootstocks of different plants. Thirteen flavonoid and phenolic compounds, 3 catechin compounds and 4 procyanidin compounds were determined and thoroughly analyzed in this study. The study outcomes related with the amounts of antioxidants and other important substances in grafted plants (not only woods) may provoke future studies on the subject prior to an elucidation of the other compounds. Additionally, novel biochemical studies aimed at elucidating the biochemical mechanisms of affinities during grafting may also be done based on the clues revealed here in the present study.

## Figures and Tables

**Table 1:** Parameters characterizing LC/MS detection for flavonoid compounds.

Compound Name	Precursor Ion	Product Ion	Fragmentation Voltage [V]	Collision Energy [V]	Polarity
**Dihydrokaempherol**	287	259	130	4	Negative
**Eriodictyol**	287	151	106	0	Negative
Hyperoside * (Q-3-galactoside)	463	300	150	20	Negative
Isoquercitrin ** (Q-3-glucoside)	255	119	100	16	Negative
**Isovitexin**	431.1	311	140	20	Negative
**Naringeninchalcone**	271	151	104	4	Negative
**Pentahydroxychalcone**	287	151	96	8	Negative
**Quercetin**	301	151	208	8	Negative
Quercitrin *** (Q-3-rhamnoside)	447.1	300	158	16	Negative
Rutin **** (Q-3-rutinoside)	609	300	220	35	Negative
**Vitexin**	431.1	311	142	20	Negative
**Vitexin-2-O-rhamnoside**	431	268	170	32	Negative

* = Quercetin 3-D-galactoside; ** = Quercetin 3-β-D-glucoside; *** = Quercetin 3-rhamnoside; **** = Quercetin 3-rutinoside.

**Table 2:** Parameters characterizing LC/MS detection for phenolic acids and aldehydes.

Compound Name.	Precursor Ion	Product Ion	Fragmentation Voltage [V]	Collision Energy [V]	Polarity
**3,4-Dihydroxybenzaldehyde**	137	108	120	20	Negative
**Caffeic acid**	179	135	100	10	Negative
**Gallic acid**	169	125	100	10	Negative
***p*-Coumaric acid**	163	119	66	12	Negative
***p*-Hydroxybenzaldehyde**	121	92	120	20	Negative
***p*-Hydroxybenzoic acid**	137	93	100	10	Negative
**Protocatechuic acid**	153	109	100	10	Negative
**Salicylic acid**	137	93	100	10	Negative
**Syringic acid**	197	182	80	10	Negative
**Vanilic acid**	167	152	80	10	Negative
**Vanilin**	151	136	80	8	Negative
**Chlorogenic acid**	353	191	100	10	Negative
**Cryptochlorogenic acid**	353	191	105	10	Negative

**Table 3:** Parameters characterizing LC/MS detection for catechin and procyanidin compounds.

Compound Name	Precursor Ion	Product Ion	Fragmentation Voltage [V]	Collision Energy [V]	Polarity
**Catechin**	289	109	100	20	Negative
**Epicatechin**	289	245	146	4	Negative
**Epigallocatechin**	305	125	146	12	Negative
**Procyanidin a2**	575	285	170	28	Negative
**Procyanidin b1**	577.5	407	170	20	Negative
**Procyanidin b2**	577.5	407	170	16	Negative
**Procyanidin c1**	865	407	160	36	Negative

## Supplementary Materials

The following are available online at https://www.mdpi.com/2076-3921/9/3/209/s1, Table S1: Probabilities for post-hoc Dunnett’s tests (2-sided), Table S1: Correlation matrix.
