# Supplementary material for: Assessment of Antioxidants in Selected Plant Rootstocks
Source: Antioxidants (Basel). 2020 Mar 3;9(3):209. doi: 10.3390/antiox9030209 (PMC7139285; doi:10.3390/antiox9030209)
Supplement: Supplementary file 1 [file antioxidants-09-00209-s001.pdf]

Assessment of Anti-Oxidative Compounds in Selected Plant Rootstocks

Samuel Magnus [1], Filip Gazdik [2], Naser A. Anjum[3], Eliska Kadlecova [2], Zuzana Lackova [4,8], Natalia Cernei [4,8], Martin Brtnicky[5,6], Jindrich Kynicky[7], Borivoj Klejdus [4,8], Tomas Necas [1] and Ondrej Zitka [4,8,*]

1. Department of Fruit Science, Faculty of Horticulture, Mendel University in Brno, Valticka 337, 691 44 Lednice, Czech Republic; xmagnus@node.mendelu.cz (S.M.); tomas.necas@mendelu.cz (T.N.)
2. Mendeleum - Institute of Genetics, Mendel University in Brno, Valticka 334, 691 44 Lednice, Czech Republic; filip.gazdik@mendelu.cz (F.G.); xkadleco@node.mendelu.cz (E.K.)
3. Department of Botany, Aligarh Muslim University, Aligarh, 202 002, U.P., India; anjum@ua.pt (N.A.A.)
4. Department of Chemistry and Biochemistry, Mendel University in Brno, Zemedelska 1, Brno CZ-613 00, Czech Republic; zuzana.lackova@mendelu.cz (Z.L.); cernei.natalia3@gmail.com (N.C.); klejdusb@seznam.cz (B.K.); ondrej.zitka@mendelu.cz (O.Z.)
5. Department of Agrochemistry, Soil Science, Microbiology and Plant Nutrition, Mendel University, Brno, Czech Republic; Martin.Brtnicky@seznam.cz (M.B.);
6. Institute of Chemistry and Technology of Environmental Protection, Brno University of Technology, Faculty of Chemistry, Purkynova 118, 621 00 Brno, Czech Republic
7. BIC Brno, Technology Innovation Transfer Chamber, 61200, Brno, Czech Republic; jindrak@email.cz (J.K.).
8. Central European Institute of Technology, Mendel University in Brno, Zemedelska 1, Brno CZ-613 00, Czech Republic

* Correspondence: ondrej.zitka@mendelu.cz; Tel.: +420-5-4513-3350; Fax: +420-5-4521-2044

Below are showed tables of results from post-hoc Dunnett's test and correlation analysis. All statistics was done in STATISTICA CZ Version 12.0 (StatSoft CR s.r.o., Prague, Czech Republic).

Table S1. Probabilities for post-hoc Dunnett's tests (2-sided). Highlighted values are not significant (p>0.05), that means they are not statistically different from control sample.

	caffeic acid	catechin	chlorogenic acid	cryptochlorogenic acid	dihydrokaempferol	epicatechin	epigalocatechin	eriodictyol	gallic acid	homoeriodictyol	hyperoside (Q-3-galactoside	isoquercitrin (Q-3-glucoside)	isovitexin	naringenin chalcone	p-coumaric acid	pentahydroxychalcone	p-hydroxybenzaldehyde	p-hydroxybenzoic acid	procyanidin a2	procyanidin b1	procyanidin b2	procyanidin c1	protocatechuic acid	quercetin	quercitrin (Q-3-rhamnoside)	rutin (Q-3-rutinoside)	salicylic acid	syringic acid	vanilin	vanillic acid	vitexin	vitexin-2-O-rhamnoside	3,4-dihydroxybenzaldehyde
Control	x	x	x	x	x	x	x	x	x	x	x	x	x	x	x	x	x	x	x	x	x	x	x	x	x	x	x	x	x	x	x	x	x
Ba 29 (O-LE-14)	**	**	**	**	**	**	**	**	**	*	**	**	**	**	**	**	**	**	**	**	**	**	**	0.322	**	**	**	**	**	**	**	**	**
Ba 29 (O-LE-21)	**	**	**	**	1.000	**	**	**	**	**	**	**	**	**	**	**	**	**	**	**	**	**	**	**	**	**	**	**	**	**	**	**	**
Crataegus l. (O-LE-14)	**	**	**	**	0.216	**	**	**	**	**	**	**	**	**	**	**	**	**	**	**	**	**	**	**	**	**	**	**	**	**	**	**	**
Crataegus l. (O-LE-21)	**	**	**	**	**	**	**	**	**	**	**	**	**	**	**	**	**	**	**	**	**	**	**	*	**	**	**	**	**	**	**	**	**
Aronia m. (O-LE-14)	**	**	**	**	0.819	**	**	**	**	**	**	**	**	**	**	**	**	**	**	**	**	**	**	**	**	**	**	**	**	**	**	**	**
Aronia m. (O-LE-21)	**	**	**	**	**	**	**	**	**	**	**	**	**	**	**	**	**	**	**	**	**	**	**	**	**	**	**	**	**	**	**	**	**
Chaenomeles j. (O-LE-9)	**	**	**	**	**	**	**	**	**	**	**	**	**	**	**	**	**	**	**	**	**	**	**	**	**	**	**	**	**	**	**	**	**

**p<<0.001; *p<0.05; (x) not applicable

Table S2. Correlation matrix. Correlation was calculated for all variables among all samples (N=8). Highlighted correlations are significant ($p<0.05$).

	3,4-dihydroxybenzaldehyde	caffeic acid	gallic acid	p-coumaric acid	p-hydroxybenzaldehyde	p-hydroxybenzoic acid	protocatechuic acid	salicylic acid	syringic acid	vanillic acid	vanilin	chlorogenic acid	cryptochlorogenic acid	catechin	epicatechin	epigalocatechin	procyanidin a2	procyanidin b1	procyanidin b2	procyanidin c1	dihydrokaempferol	eriodictyol	homoeriodictyol	hyperoside (Q-3-galactoside)	isoquercitrin (Q-3-glucoside)	isovitexin	naringenin chalcone	pentahydroxychalcone	quercetin	quercitrin (Q-3-rhamnoside)	rutin (Q-3-rutinoside)	vitexin	vitexin-2-O-rhamnoside
3,4-dihydroxybenzaldehyde	1.000	-0.428	-0.163	-0.488	-0.122	-0.033	0.526	0.852	-0.383	-0.345	-0.300	0.964	0.954	0.972	0.903	0.713	-0.233	0.936	0.657	0.506	0.086	-0.128	0.755	-0.002	0.082	-0.125	0.764	-0.128	0.755	-0.054	-0.263	-0.090	-0.218
caffeic acid	-0.428	1.000	0.466	0.819	0.878	0.863	0.392	-0.643	-0.204	-0.109	-0.332	-0.322	-0.308	-0.509	-0.435	-0.557	-0.051	-0.584	-0.116	-0.445	-0.382	0.249	-0.494	0.097	0.043	-0.440	-0.329	0.249	-0.494	0.732	0.756	-0.457	-0.219
gallic acid	-0.163	0.466	1.000	0.723	0.191	0.233	0.288	-0.106	-0.124	-0.140	-0.025	-0.098	-0.098	-0.199	-0.213	-0.474	-0.322	-0.265	-0.067	-0.472	0.470	0.705	-0.158	-0.351	-0.336	-0.269	0.207	0.705	-0.158	0.298	0.293	-0.298	-0.513
p-coumaric acid	-0.488	0.819	0.723	1.000	0.542	0.517	0.385	-0.500	0.026	0.051	0.018	-0.414	-0.353	-0.607	-0.555	-0.737	-0.044	-0.621	-0.141	-0.409	0.019	0.692	-0.448	-0.019	-0.075	-0.297	-0.388	0.692	-0.448	0.390	0.531	-0.312	-0.177
p-hydroxybenzaldehyde	-0.122	0.878	0.191	0.542	1.000	0.992	0.446	-0.539	-0.241	-0.084	-0.414	-0.076	-0.093	-0.206	-0.225	-0.302	-0.088	-0.304	0.080	-0.244	-0.666	0.046	-0.450	0.154	0.113	-0.437	-0.191	0.046	-0.450	0.683	0.573	-0.449	-0.188
p-hydroxybenzoic acid	-0.033	0.863	0.233	0.517	0.992	1.000	0.501	-0.451	-0.334	-0.175	-0.491	0.020	0.001	-0.113	-0.129	-0.259	-0.169	-0.232	0.106	-0.255	-0.631	0.054	-0.361	0.097	0.066	-0.499	-0.089	0.054	-0.361	0.710	0.578	-0.511	-0.284
protocatechuic acid	0.526	0.392	0.288	0.385	0.446	0.501	1.000	0.391	-0.508	-0.466	-0.477	0.638	0.688	0.409	0.411	0.119	-0.100	0.345	0.558	0.144	0.038	0.269	0.394	0.308	0.316	-0.482	0.369	0.269	0.394	0.473	0.413	-0.438	-0.145
salicylic acid	0.852	-0.643	-0.106	-0.500	-0.539	-0.451	0.391	1.000	-0.361	-0.424	-0.190	0.861	0.883	0.859	0.852	0.637	-0.196	0.846	0.471	0.434	0.478	-0.030	0.915	-0.043	0.032	-0.060	0.721	-0.030	0.915	-0.255	-0.329	-0.016	-0.145
syringic acid	-0.383	-0.204	-0.124	0.026	-0.241	-0.334	-0.508	-0.361	1.000	0.979	0.968	-0.543	-0.516	-0.453	-0.373	0.001	0.645	-0.135	0.149	0.461	0.042	0.041	-0.369	0.142	0.173	0.886	-0.472	0.041	-0.369	-0.347	-0.188	0.867	0.515
vanillic acid	-0.345	-0.109	-0.140	0.051	-0.084	-0.175	-0.466	-0.424	0.979	1.000	0.918	-0.528	-0.503	-0.436	-0.359	-0.010	0.587	-0.131	0.176	0.471	-0.128	0.037	-0.418	0.098	0.130	0.833	-0.497	0.037	-0.418	-0.289	-0.162	0.810	0.436
vanilin	-0.300	-0.332	-0.025	0.018	-0.414	-0.491	-0.477	-0.190	0.968	0.918	1.000	-0.464	-0.424	-0.370	-0.304	-0.004	0.549	-0.062	0.143	0.458	0.233	0.185	-0.239	0.038	0.076	0.865	-0.376	0.185	-0.239	-0.480	-0.304	0.849	0.441

chlorogenic acid	0.964	-0.322	-0.098	-0.414	-0.076	0.020	0.638	0.861	-0.543	-0.528	-0.464	1.000	0.986	0.957	0.901	0.695	-0.213	0.887	0.642	0.395	0.172	-0.172	0.789	0.115	0.189	-0.247	0.826	-0.172	0.789	0.117	-0.090	-0.204	-0.196
cryptochlorogenic acid	0.954	-0.308	-0.098	-0.353	-0.093	0.001	0.688	0.883	-0.516	-0.503	-0.424	0.986	1.000	0.918	0.906	0.663	-0.194	0.873	0.657	0.451	0.177	-0.100	0.840	0.111	0.184	-0.241	0.750	-0.100	0.840	0.090	-0.064	-0.193	-0.194
catechin	0.972	-0.509	-0.199	-0.607	-0.206	-0.113	0.409	0.859	-0.453	-0.436	-0.370	0.957	0.918	1.000	0.889	0.738	-0.271	0.921	0.552	0.395	0.144	-0.239	0.736	-0.011	0.069	-0.151	0.841	-0.239	0.736	-0.072	-0.318	-0.118	-0.206
epicatechin	0.903	-0.435	-0.213	-0.555	-0.225	-0.129	0.411	0.852	-0.373	-0.359	-0.304	0.901	0.906	0.889	1.000	0.828	-0.111	0.922	0.644	0.568	0.159	-0.327	0.908	-0.008	0.104	-0.006	0.741	-0.327	0.908	0.092	-0.040	0.029	-0.245
epigalocatechin	0.713	-0.557	-0.474	-0.737	-0.302	-0.259	0.119	0.637	0.001	-0.010	-0.004	0.695	0.663	0.738	0.828	1.000	0.361	0.889	0.738	0.744	0.142	-0.672	0.676	0.355	0.472	0.420	0.629	-0.672	0.676	0.086	-0.052	0.456	0.239
procyanidin a2	-0.233	-0.051	-0.322	-0.044	-0.088	-0.169	-0.100	-0.196	0.645	0.587	0.549	-0.213	-0.194	-0.271	-0.111	0.361	1.000	0.049	0.474	0.575	0.116	-0.431	-0.094	0.794	0.827	0.762	-0.225	-0.431	-0.094	0.191	0.323	0.789	0.853
procyanidin b1	0.936	-0.584	-0.265	-0.621	-0.304	-0.232	0.345	0.846	-0.135	-0.131	-0.062	0.887	0.873	0.921	0.922	0.889	0.049	1.000	0.765	0.698	0.213	-0.305	0.783	0.127	0.241	0.204	0.756	-0.305	0.783	-0.078	-0.248	0.240	-0.010
procyanidin b2	0.657	-0.116	-0.067	-0.141	0.080	0.106	0.558	0.471	0.149	0.176	0.143	0.642	0.657	0.552	0.644	0.738	0.474	0.765	1.000	0.797	0.141	-0.206	0.489	0.498	0.606	0.379	0.495	-0.206	0.489	0.303	0.216	0.419	0.251
procyanidin c1	0.506	-0.445	-0.472	-0.409	-0.244	-0.255	0.144	0.434	0.461	0.471	0.458	0.395	0.451	0.395	0.568	0.744	0.575	0.698	0.797	1.000	0.001	-0.308	0.506	0.371	0.469	0.663	0.126	-0.308	0.506	-0.135	-0.084	0.700	0.407
dihydrokaempferol	0.086	-0.382	0.470	0.019	-0.666	-0.631	0.038	0.478	0.042	-0.128	0.233	0.172	0.177	0.144	0.159	0.142	0.116	0.213	0.141	0.001	1.000	0.245	0.378	0.023	0.075	0.221	0.468	0.245	0.378	-0.130	-0.067	0.235	0.060
eriodictyol	-0.128	0.249	0.705	0.692	0.046	0.054	0.269	-0.030	0.041	0.037	0.185	-0.172	-0.100	-0.239	-0.327	-0.672	-0.431	-0.305	-0.206	-0.308	0.245	1.000	-0.166	-0.460	-0.500	-0.292	-0.183	1.000	-0.166	-0.272	-0.162	-0.309	-0.396
homoeriodictyol	0.755	-0.494	-0.158	-0.448	-0.450	-0.361	0.394	0.915	-0.369	-0.418	-0.239	0.789	0.840	0.736	0.908	0.676	-0.094	0.783	0.489	0.506	0.378	-0.166	1.000	-0.026	0.065	-0.025	0.600	-0.166	1.000	-0.042	-0.037	0.020	-0.196
hyperoside (Q-3-galactoside)	-0.002	0.097	-0.351	-0.019	0.154	0.097	0.308	-0.043	0.142	0.098	0.038	0.115	0.111	-0.011	-0.008	0.355	0.794	0.127	0.498	0.371	0.023	-0.460	-0.026	1.000	0.987	0.275	-0.006	-0.460	-0.026	0.370	0.381	0.325	0.842
isoquercitrin (Q-3-glucoside)	0.082	0.043	-0.336	-0.075	0.113	0.066	0.316	0.032	0.173	0.130	0.076	0.189	0.184	0.069	0.104	0.472	0.827	0.241	0.606	0.469	0.075	-0.500	0.065	0.987	1.000	0.356	0.088	-0.500	0.065	0.396	0.396	0.407	0.814
isovitexin	-0.125	-0.440	-0.269	-0.297	-0.437	-0.499	-0.482	-0.060	0.886	0.833	0.865	-0.247	-0.241	-0.151	-0.006	0.420	0.762	0.204	0.379	0.663	0.221	-0.292	-0.025	0.275	0.356	1.000	-0.136	-0.292	-0.025	-0.241	-0.133	0.997	0.568
naringenin chalcone	0.764	-0.329	0.207	-0.388	-0.191	-0.089	0.369	0.721	-0.472	-0.497	-0.376	0.826	0.750	0.841	0.741	0.629	-0.225	0.756	0.495	0.126	0.468	-0.183	0.600	-0.006	0.088	-0.136	1.000	-0.183	0.600	0.200	-0.069	-0.119	-0.276

pentahydroxychalcone	-0.128	0.249	0.705	0.692	0.046	0.054	0.269	-0.030	0.041	0.037	0.185	-0.172	-0.100	-0.239	-0.327	-0.672	-0.431	-0.305	-0.206	-0.308	0.245	1.000	-0.166	-0.460	-0.500	-0.292	-0.183	1.000	-0.166	-0.272	-0.162	-0.309	-0.396
quercetin	0.755	-0.494	-0.158	-0.448	-0.450	-0.361	0.394	0.915	-0.369	-0.418	-0.239	0.789	0.840	0.736	0.908	0.676	-0.094	0.783	0.489	0.506	0.378	-0.166	1.000	-0.026	0.065	-0.025	0.600	-0.166	1.000	-0.042	-0.037	0.020	-0.196
quercitrin (Q-3-rhamnoside)	-0.054	0.732	0.298	0.390	0.683	0.710	0.473	-0.255	-0.347	-0.289	-0.480	0.117	0.090	-0.072	0.092	0.086	0.191	-0.078	0.303	-0.135	-0.130	-0.272	-0.042	0.370	0.396	-0.241	0.200	-0.272	-0.042	1.000	0.922	-0.236	-0.107
rutin (Q-3-rutinoside)	-0.263	0.756	0.293	0.531	0.573	0.578	0.413	-0.329	-0.188	-0.162	-0.304	-0.090	-0.064	-0.318	-0.040	-0.052	0.323	-0.248	0.216	-0.084	-0.067	-0.162	-0.037	0.381	0.396	-0.133	-0.069	-0.162	-0.037	0.922	1.000	-0.124	-0.016
vitexin	-0.090	-0.457	-0.298	-0.312	-0.449	-0.511	-0.438	-0.016	0.867	0.810	0.849	-0.204	-0.193	-0.118	0.029	0.456	0.789	0.240	0.419	0.700	0.235	-0.309	0.020	0.325	0.407	0.997	-0.119	-0.309	0.020	-0.236	-0.124	1.000	0.605
vitexin-2-O-rhamnoside	-0.218	-0.219	-0.513	-0.177	-0.188	-0.284	-0.145	-0.145	0.515	0.436	0.441	-0.196	-0.194	-0.206	-0.245	0.239	0.853	-0.010	0.251	0.407	0.060	-0.396	-0.196	0.842	0.814	0.568	-0.276	-0.396	-0.196	-0.107	-0.016	0.605	1.000
